# DARPP-32 Is a Robust Integrator of Dopamine and Glutamate Signals

**DOI:** 10.1371/journal.pcbi.0020176

**Published:** 2006-12-22

**Authors:** Éric Fernandez, Renaud Schiappa, Jean-Antoine Girault, Nicolas Le Novère

**Affiliations:** 1 EMBL–EBI, Wellcome-Trust Genome Campus, Hinxton, United Kingdom; 2 INSERM U536, Institut du Fer a Moulin, Paris, France; 3 Université de Pierre et Marie Curie; University College London, United Kingdom

## Abstract

Integration of neurotransmitter and neuromodulator signals in the striatum plays a central role in the functions and dysfunctions of the basal ganglia. DARPP-32 is a key actor of this integration in the GABAergic medium-size spiny neurons, in particular in response to dopamine and glutamate. When phosphorylated by cAMP-dependent protein kinase (PKA), DARPP-32 inhibits protein phosphatase-1 (PP1), whereas when phosphorylated by cyclin-dependent kinase 5 (CDK5) it inhibits PKA. DARPP-32 is also regulated by casein kinases and by several protein phosphatases. These complex and intricate regulations make simple predictions of DARPP-32 dynamic behaviour virtually impossible. We used detailed quantitative modelling of the regulation of DARPP-32 phosphorylation to improve our understanding of its function. The models included all the combinations of the three best-characterized phosphorylation sites of DARPP-32, their regulation by kinases and phosphatases, and the regulation of those enzymes by cAMP and Ca^2+^ signals. Dynamic simulations allowed us to observe the temporal relationships between cAMP and Ca^2+^ signals. We confirmed that the proposed regulation of protein phosphatase-2A (PP2A) by calcium can account for the observed decrease of Threonine 75 phosphorylation upon glutamate receptor activation. DARPP-32 is not simply a switch between PP1-inhibiting and PKA-inhibiting states. Sensitivity analysis showed that CDK5 activity is a major regulator of the response, as previously suggested. Conversely, the strength of the regulation of PP2A by PKA or by calcium had little effect on the PP1-inhibiting function of DARPP-32 in these conditions. The simulations showed that DARPP-32 is not only a robust signal integrator, but that its response also depends on the delay between cAMP and calcium signals affecting the response to the latter. This integration did not depend on the concentration of DARPP-32, while the absolute effect on PP1 varied linearly. In silico mutants showed that Ser137 phosphorylation affects the influence of the delay between dopamine and glutamate, and that constitutive phosphorylation in Ser137 transforms DARPP-32 in a quasi-irreversible switch. This work is a first attempt to better understand the complex interactions between cAMP and Ca^2+^ regulation of DARPP-32. Progressive inclusion of additional components should lead to a realistic model of signalling networks underlying the function of striatal neurons.

## Synopsis

Projecting neurons of the striatum are a crucial relay of the basal ganglia, involved in motor, psychomotor, and behavioural functions. Their importance is emphasised by their involvement in various dysfunctions, such as Huntington chorea and schizophrenia, but also drug addiction. The main inputs to those neurons come from cortical glutamatergic terminals. Dopamine modulates this transmission, providing a measure of the internal (hedonic) state. In mammal brain, DARPP-32, a protein phosphatase inhibitor, has been identified as a major target for both dopamine and glutamate signalling. The authors present a detailed quantitative model of the regulation of DARPP-32 phosphorylation and dephosphorylation by both signals. Dynamic simulations show that the function of DARPP-32 depends on the delay between the two signals, and therefore the protein not only measures the intensity, but also the coincidence, between signals. This measurement is insensitive to many parameters, whether kinetic constants or concentrations, making it a robust integrator. This shows that a proper understanding of signal integration in the basal ganglia requires quantitative descriptions of the signalling pathways in addition to the neuronal electrophysiological properties.

## Introduction

The basal ganglia of mammals are made up of several nuclei forming large processing circuits in the forebrain and controlled by mesencephalic dopamine (DA) neurons [1]. The dorsal nigrostriatal DA pathway modulates the cortico–striato–thalamic loop [2] involved in extrapyramidal motor and cognitive functions. The ventral mesolimbic DA pathway supports a variety of behavioural functions related to motivation and reward [3]. The functional diversity of the basal ganglia is mirrored by their involvement in pathological conditions as diverse as Parkinson disease, Huntington chorea, schizophrenic syndromes, and drug addiction. The main inputs of the striatum are the excitatory glutamatergic projections from pyramidal neurons of the cortex [4,5]. The GABAergic medium-sized spiny neurons, which comprise more than 95% of the striatal neurons, give rise to two kinds of projections. A direct “stimulatory” pathway projects to the output structures, internal globus pallidus, and substantia nigra pars reticulata, while an indirect, “depressant” pathway projects to the same nuclei via the external globus pallidus and the subthalamic nucleus [6]. The indirect pathway forms an incoherent feedforward loop (that is in the same direction as the direct pathway but with opposite effect), that modulates the effect of the direct pathway. The balance between those two pathways is crucial for the function of basal ganglia. DA released in striatum potentiates the function of the direct pathway, through D1 receptors, and acts as a psychostimulant (enhancing locomotion and elevating mood). In addition, DA inhibits the function of the indirect pathway through D2 receptors. The disappearance of this control contributes to the clinical symptoms of Parkinson disease.

**Figure 1:**
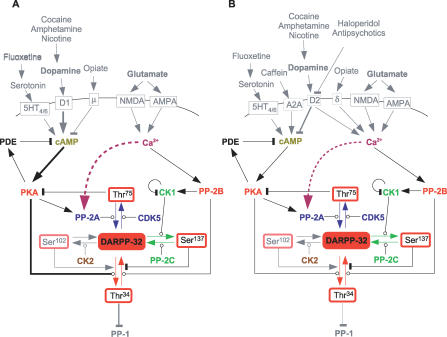
Biological Model of DARPP-32 Regulation The various endogenous external signals affecting DARPP-32 through cAMP and calcium are represented, as well as external drugs. (A) nigro-striatal medium-sized spiny GABAergic neuron; (B) nigro-pallidal medium-size spiny GABAergic neuron. Arrow-ending lines represent stimulation, bar-ending lines represent inhibition, circle-ending lines represent enzymatic reactions. Dashed lines represent reactions only present in model B. Source is [9].

A key actor in the integration of DA and glutamate is DARPP-32, the dopamine and cAMP-regulated phosphoprotein of 32 kDa (Figure 1). DARPP-32 is a protein phosphatase inhibitor similar to inhibitor protein 1, highly expressed in medium-sized spiny neurons of the neostriatum [7–9]. It has been initially identified as a major target for DA signalling in striatal neurons [10,11]. However, subsequent studies have shown that DARPP-32 plays a wider role in the integration of numerous signals arriving at dopaminoceptive neurons [12,13].

DARPP-32 is phosphorylated on Thr34 by cAMP-dependent protein kinase (PKA) upon activation of the cAMP signalling pathway, for instance, by dopamine via the D1 receptors (Figure 1). This phosphorylated form (D34) acts as a potent inhibitor of protein phosphatase-1 (PP1). PP1 affects many signalling steps, by dephosphorylating receptors such as AMPA and NMDA glutamate receptors, or GABA_A_ receptors, voltage-gated ion channels (Na^2+^, L-, and N/P-Ca^2+^), kinases such as calcium/calmodulin kinase II, and transcription factors (e.g., CREB), etc. (see [9] for a review). Dephosphorylation of Thr34 is mediated by protein phosphatase-2B (PP2B, also called calcineurin), upon activation of the Ca^2+^ pathway. Contrarily to inhibitor protein 1, DARPP-32 possesses several other phosphorylation sites that modulate its ability to inhibit PP1. DARPP-32 is phosphorylated on Thr75 by the cell division protein kinase 5 (CDK5) [14]. This phosphorylated form (D75) acts as an inhibitor of PKA, thus reducing the effect of dopamine signalling on PKA targets such as AMPA glutamate receptors, MAPKKK, CREB, etc. D75 dephosphorylation is enhanced by PKA via the activation of protein phosphatase-2A (PP2A), forming a positive feedback loop [15]. Interestingly, activation of the Ca^2+^ pathway also leads to dephosphorylation of Thr75 [16]. The biochemical basis of this effect is still unclear. It could be mediated by the phosphorylation of the B′ PP2A regulatory subunit by calcium/calmodulin kinase II [17] or via the interaction of PP2A with the calmodulin-binding protein striatin [18]. One can also hypothesize more indirect mechanisms, such as the release of PP2A catalytic subunits by CaMKIV, upon binding of Ca^2+^/calmodulin [19]. The effect of calcium therefore creates an incoherent feedforward loop—the Ca^2+^ at the same time activating the phosphorylation on Thr34 via the relief of PKA inhibition, and activating the dephosphorylation of Thr34 via the activation of PP2B.

It has therefore been suggested that DARPP-32 functioned as a molecular switch, acting either as a PKA inhibitor or PP1 inhibitor. The switch would be controlled by the activity of two major signalling pathways: cAMP/PKA/D34 and Ca^2+^/PP2B/D75. By modulating the activity of these two pathways, it has been shown that DARPP-32 played a critical role in the function of the cortico–striato–thalamo-cortical loop, both in response to glutamatergic, GABAergic, and dopaminergic inputs, but also to therapeutical drugs and drugs of abuse [20,21]. In particular, phosphorylation of DARPP-32 on Thr75 is a crucial factor in the sensitivity to dopamine, greatly affected by cocaine treatment, through the modulation of CDK5 activity [22].

Two other phosphorylation sites modulate DARPP-32 function. Under basal conditions, DARPP-32 is phosphorylated on Ser102 and Ser137, by casein kinase 2 and 1 (CK2 and CK1), respectively [23,24]. These phosphorylations in turn modulate the phosphorylation of DARPP-32 at Thr34. Phosphorylation on Ser137 decreases the rate of Thr34 dephosphorylation by PP2B, whereas phosphorylation on Ser102 increases the rate of phosphorylation on Thr34 by PKA. The activity of CK1 is suppressed by its (auto)phosphorylation. PP2B dephosphorylates CK1, and therefore enhances the phosphorylation of Ser137 [25], forming another incoherent feedforward loop, the Ca^2+^ at the same time activating and inhibiting the dephosphorylation of Thr34. Ser137 is dephosphorylated by protein phosphatase-2C (PP2C).

Paradoxically, the knowledge acquired about the regulation of DARPP-32 function in the medium-sized spiny neurons of the striatum is sufficiently detailed to make simple “pen and paper” predictions of the behaviour of the whole system a very difficult, if not impossible, task. DARPP-32 effectively represents a “hub” connecting at least four kinases and three phosphatases. However, because of the differential effects of each phosphorylation on the regulation of the others, the final picture is that of a whole signalling network made up of one protein. Due to the numerous elementary activation and inhibition steps, or positive and negative feedback and feedforward loops identified, contradictions quickly emerge when we try to consider all the possible reactions. Numerical simulations are therefore mandatory to gather quantitative descriptions and test various hypotheses proposed in the literature. Previous attempts have been made to develop quantitative models of DARPP-32 function, but only considered phosphorylation on Thr34 [26,27]. Lindskog and colleagues [28] also considered phosphorylation on Thr75 (but none of the serine phosphorylations).

We constructed a computational model of DARPP-32 phosphorylation. The model also included the regulation of kinases and phosphatases by cAMP and Ca^2+^ signals. The model reproduced key behaviours experimentally observed, such as dopamine and glutamate effects on Thr34 phosphorylation, and sensitivity of the response of CDK5 activity to cAMP. We also implemented the regulation of PP2A activity by calcium, suggested by Nishi et al. [16], and show that surprisingly the noticeable effect on Thr75 phosphorylation has very minor consequences for PKA activity and Thr34 phosphorylation. In addition, the models describe several behaviours that should be experimentally testable.

## Results

### Model Construction

**Figure 2:**
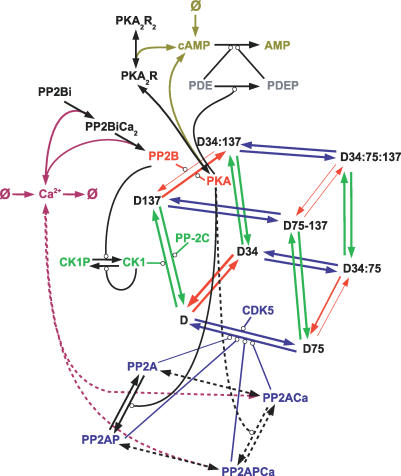
Biochemical Model of DARPP-32 Regulation Graphical representation of the models implemented in this study. Arrow-ending lines represent transition, either phosphorylations or binding. Note that the bindings are reversible. Circle-ending lines represent enzymatic reactions. The effects of kinases and phosphatases on DARPP-32 have been represented only once for clarity, but each couple of enzymes effectively acts on every pair of arrows of the same colour. The different thicknesses of the red-arrowed lines represent the catalytic rates for the various DARPP-32 species. Dashed lines represent reactions only present in model B. Colour code of the molecular species is the same as for Figure 1.

The core of our model was centred on three phosphorylation sites of DARPP-32: threonine 34, threonine 75, and serine 137. Therefore, our DARPP-32 molecule can present eight phosphorylation states, from the unphosphorylated to the triply phosphorylated form (Figure 2). The transitions between the various phosphorylation states corresponded to 12 phosphorylation reactions and 12 dephosphorylation reactions, catalyzed by three kinases (CDK5, CK1, PKA) and three phosphatases (PP2A, PP2B and PP2C). The phosphorylation of serine 102 by CK2, which has a relatively small effect on phosphorylation by PKA [23], was discarded from the current version of our models. Since neither cAMP nor calcium signals affect its phosphorylation [9], its presence or absence would not modify the behaviour of the model upon perturbations.

**Figure 3:**
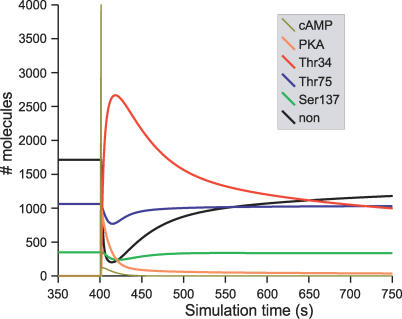
Effect of a Pulse of cAMP on DARPP-32 Phosphorylation Time-course of DARPP-32 isoforms after a pulse of cAMP. Brown line represents the number of cAMP molecules. Orange line represents the total number of PKA catalytic subunits not bound to regulatory subunits. DARPP-32 species are represented in black (unphosphorylated), red (D34*), blue (D75*), and green (D137*).

PKA is a four-subunit enzyme, composed of two regulatory subunits and two catalytic subunits, and its regulation was modelled as previously described [29]. In these models, each regulatory subunit of PKA can bind two cAMP molecules, which leads to the release of the catalytic subunits, representing the active PKA molecules. cAMP was degraded into AMP by phosphodiesterase (PDE), which was itself activated by PKA [30]. Ca^2+^, produced, for instance, by the activation of NMDA receptors, was modelled as a controlled fixed influx with a basal rate, changed upon perturbation, and an efflux depending on the concentration of Ca^2+^. Note that in our model, adenylyl cyclase was not Ca^2+^-sensitive, and therefore cAMP and PKA were neither activated nor inhibited by Ca^2+^. Although we are aware that adenylyl cyclase V, abundantly expressed in striatum, is inhibited by Ca^2+^ [31,32], our focus was the integration of cAMP and Ca^2+^ signals at the level of DARPP-32.

The activation of PP2B was modelled following a simplified scheme by binding two pairs of Ca^2+^ ions to an inactive form of the enzyme without explicit representation of calmodulin.

CK1 is a ubiquitous serine protein kinase in eukaryotic organisms, which targets a wide range of substrates and participates in a large number of processes [33]. “CK1” is actually a protein family including at least seven isoforms in mammals and their various splice variants. Isoforms and variants display different kinase activity and tissue expression or subcellular localization. Isoforms alpha, delta, and epsilon are expressed in neurons of the striatum [25,34,35]. Interestingly, isoforms delta and epsilon are subject to inhibitory autophosphorylation on the COOH-terminal regulatory domain [36–38]. To analyse the level of this auto-inactivation and its influence on the signal integration by DARPP-32, we included an autophosphorylation of CK1 in our model.

The model also included the following four modulations. 1) DARPP-32 phosphorylated on Thr75 (D75, D75:137) were very poor substrates for PKA, resulting in a competitive inhibition [14]. Since our models assume an absence of product rebinding, the forms phosphorylated on Thr34 do not bind to PKA, and the multiple phosphorylations D34:75 and D34:75:137 do not inhibit PKA. To account for the fact that D75* are also poor substrates for PP2B [15,39], the dissociation constant of D75* with PP2B was increased, assuming that phosphorylation was impairing the association between the two molecules. 2) all four forms of DARPP-32 phosphorylated on Ser137 (D137, D34:137, D75:137, D34:75:137, collectively named D137*) were poor substrates for PP2B [24]. This was translated in the model by modified association, dissociation, and catalytic constants of reactions performed by PP2B on D137*. 3) PP2A was stimulated upon phosphorylation by PKA [15]. 4) CK1 was activated upon dephosphorylation by PP2B [36].

One of the major questions about the DARPP-32 role as an integrator of signals, is the observed dephosphorylation of Thr75 following activation of NMDA and AMPA receptors. As suggested by Nishi et al. [16], dephosphorylation of Thr75 could be mediated through Ca^2+^-dependent activation of PP2A. We therefore constructed model B, identical to model A, but including binding of Ca^2+^ on PP2A, formation of a catalytically more active form of PP2A, and therefore stimulation of Thr75 dephosphorylation activity (dotted arrows on Figure 2).

All elementary reactions are listed in Table 1. The enzymatic processes were decomposed into three elementary steps, without any assumptions of equilibrium or steady-state (see Discussion). Quantitative parameters were extracted from literature or databases, or estimated, so that basal conditions at the equilibrium matched concentrations of the various DARPP-32 species observed in vivo [9]. The values of the parameters we used are listed in Table 1 (except for sensitivity analysis, in which several values were tested, Figures 5, 7, and 8). All the reactions were assumed to take place in the volume of a dendritic spine, which we evaluated at 10^−15^ L [40].

**Table 1:**
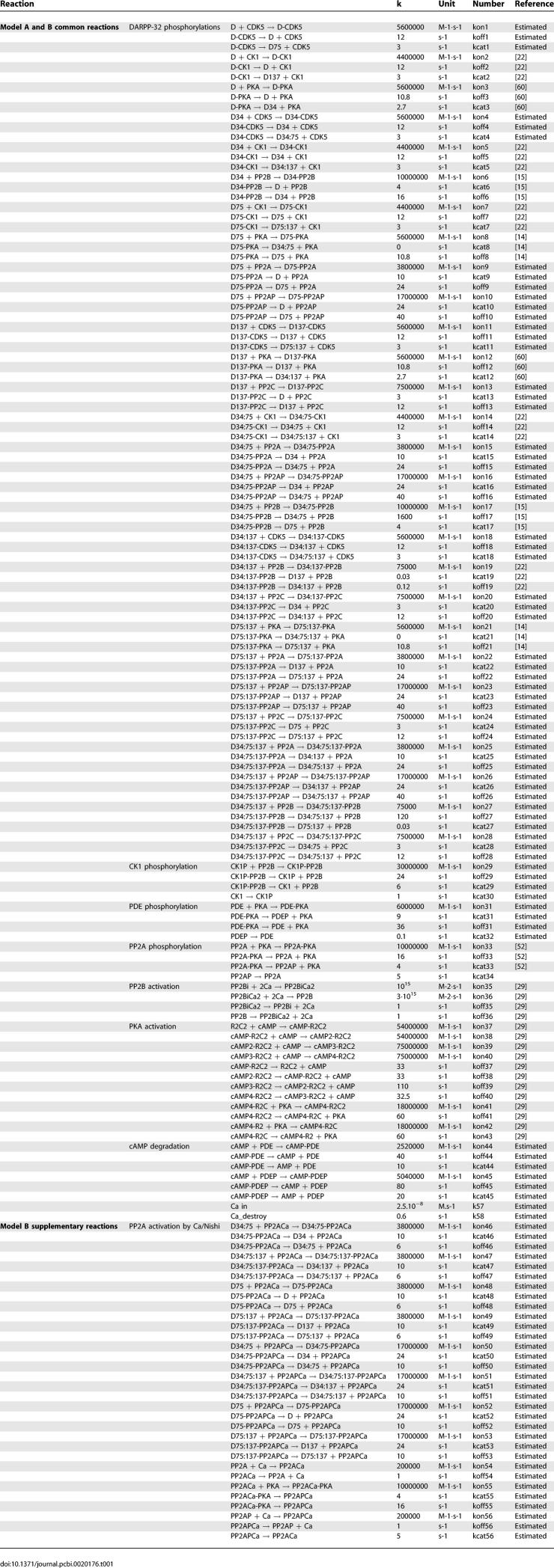
Elementary Reactions and Parameters Used in the Models

**Figure 4:**
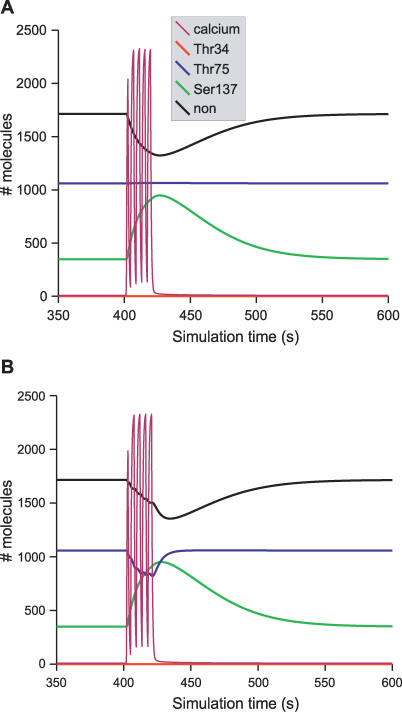
Effects of a Train of Ca^2+^ Spikes on DARPP-32 Phosphorylation Time-course of DARPP-32 isoforms triggered by a train of Ca^2+^ spikes. Bordeaux line represents the number of calcium ions in the dendritic spine. Colour code of DARPP-32 isoforms is the same as for Figure 3. In the absence of a cAMP signal, the phosphorylation on Thr34 remains null. (A) model A; (B) model B.

**Figure 5:**
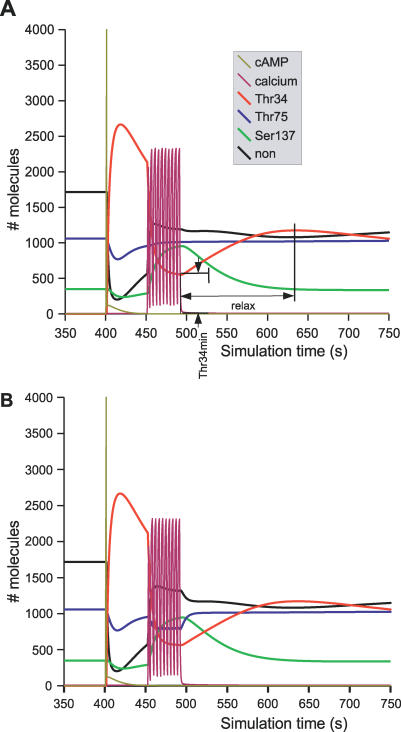
Effect of One Pulse of cAMP Followed by a Train of Ca^2+^ Spikes on DARPP-32 Phosphorylation Time-course of DARPP-32 isoforms triggered by a pulse of cAMP followed by a train of Ca^2+^ spikes. Colour code of DARPP-32 isoforms is the same as for Figure 3. Relax and Thr34min show the two readouts used in sensitivity analysis. (A) model A; (B) model B.

### Dynamic Simulation of the Models and Comparison with Experimental Results

We analysed the behaviour of our two models (model A and model B) perturbed in different ways: 1) by a pulse of cAMP, which represented the activation of adenylate cyclase by dopamine D1 receptors; 2) by a train of Ca^2+^ spikes, which represented the activation of glutamate NMDA receptors; 3) by a pulse of cAMP followed by a train of Ca^2+^ spikes, to analyse the modulation by dopamine of the response to glutamate.

Before the perturbations, the simulation was run until a stable state was reached for each molecular species. Initial conditions are listed in Table 2. Initially, in the absence of cAMP, the system did not contain any active PKA. On the contrary, free Ca^2+^ concentration reached ∼1.5 10^−8 ^M after equilibrium, and a basal activity of PP2B was therefore maintained. PP2B activity in turn maintained activated CK1. In our initial conditions, after reaching equilibrium, unphosphorylated DARPP-32 represented ∼57% of total DARPP-32, D75* ∼35%, D137* ∼13%, and D75:137 ∼4%. The total amount of DARPP-32 in the system was 3000 molecules, corresponding to a concentration of 5 × 10^−6^ M in a total volume of 10^−15 ^L.

**Table 2:**
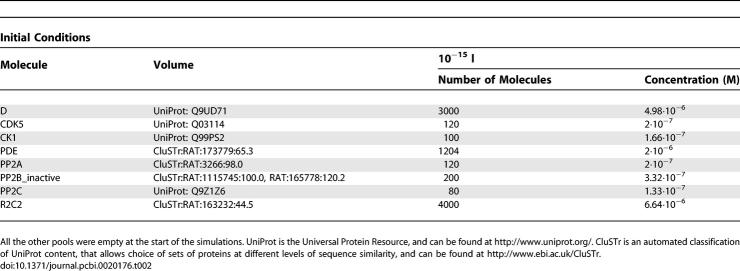
Initial Conditions

### cAMP Pulse

cAMP activation was simulated by introducing 4000 molecules of cAMP into the system (equivalent to 6.6 × 10^−6 ^M). cAMP molecules bound the regulatory subunits of PKA, which led to the dissociation of the catalytic subunits, which in turn led to phosphorylation of DARPP-32 on Thr34 (Figure 3). At the peak of activity, 1000 molecules (1.7 × 10^−6 ^M) of catalytic PKA subunits are available. D34* peaked after 20 seconds, reaching ∼89% of DARPP-32. Following the degradation of cAMP by PDE and the reassociation of the catalytic subunits of PKA with its regulatory subunits, Thr34 phosphorylation decayed exponentially, due to the basal activity of PP2B. Upon activation of the cAMP pathway, we also observed a decrease of D75* from 35% to 25%, caused by the potentiation of PP2A by active PKA. A decrease of D137* to 7.7% was caused by the segregation of PP2B by D34*, resulting in a decrease in CK1 activation and therefore a decrease of Ser137 phosphorylation. This effect was confirmed by another simulation: removing the autophosphorylation of CK1 suppressed the effect of cAMP pathway activation on Ser137 phosphorylation (unpublished data). While D75 and D137 return to their basal levels quite rapidly, after about 1 minute, only two-thirds of the D34 has disappeared after half an hour.

Since the Ca^2+^ pathway was not activated in this experiment, models A and B presented the same behaviour upon the sole activation of the cAMP pathway.

### Ca^2+^ Spikes

Activation of the Ca^2+^ pathway was performed by repeatedly increasing the influx rate of Ca^2+^ into the system, from 2.5 × 10^−8 ^Ms^−1^ to 6.6 × 10^−6 ^Ms^−1^ , every 4 s for 2 s. This triggered the formation of a series of spikes during which Ca^2+^ transiently reached a concentration of 4 × 10^−6 ^M, then decayed to its basal level. The two models A and B exhibited different behaviours upon activation of the Ca^2+^ pathway. In model A, without Ca^2+^ activation of PP2A, Ca^2+^ spikes increased Ser137 phosphorylation, up to ∼32% (Figure 4). The increase of this phosphorylation was performed by consuming unphosphorylated DARPP-32 (which dropped to 42%) or by phosphorylating D75. The total level of phosphorylation on Thr75 was found almost not modified. This result is not consistent with in vivo experiments on rats that showed a dephosphorylation of DARPP-32 on Thr75 triggered by the activation NMDA receptors. We therefore tested model B, which included the activation of PP2A by Ca^2+^ suggested by Nishi et al. [16]. Phosphorylation of DARPP-32 on Ser137 upon activation of the Ca^2+^ pathway was observed at the same rate as in model A. However, we observed a decrease of D75*, as observed in vivo. The levels of unphosphorylated DARPP-32 dropped as in model A, due to the increased phosphorylation on Ser137. However, during the calcium spikes, this decrease was counteracted by the increased dephosphorylation of D75. Overall, D137 was produced mainly from unphosphorylated DARPP-32 rather than from D75, resulting in lower D75:137 double phosphorylations (unpublished data).

### cAMP Pulse Followed by Ca^2+^ Spikes

To study the modulatory effect of D1 receptor activation on glutamate signals, we ran simulations where both cAMP and Ca^2+^ pathways were activated at different time intervals. The simulation run consisted, after reaching steady state, of a cAMP pulse followed by a train of Ca^2+^ spikes. The delay between the two activations was variable, ranging between 0 and 1000 s. This covers the short dopamine–glutamate interactions described, for instance, in Kotter and Wickens (1995) [9], but also the long lasting psychostimulation observed after dopamine has been increased, for instance, by nicotine or cocaine [41,42].

Figure 5 shows a typical result of the activation of the cAMP pathway, followed after 50 s by activation of the Ca^2+^ pathway. Using model A, the cAMP pulse led to phosphorylation of Thr34 and dephosphorylation of both Thr75 and Ser137 (Figure 5A). When the Ca^2+^ pathway was activated, increases of unphosphorylated DARPP-32 and phosphorylation of Ser137 were observed, while the level of Thr34 phosphorylation plunged, due to the activation of PP2B. The phosphorylation of Thr75 slowly increased at the end of PKA activation to reach its steady state. This phosphorylation was unaffected by the Ca^2+^ spikes in model A. Model B behaved similarly upon increase of cAMP (Figure 5B) except that D75* decreased due to the activation of PP2A by Ca^2+^.

**Figure 6:**
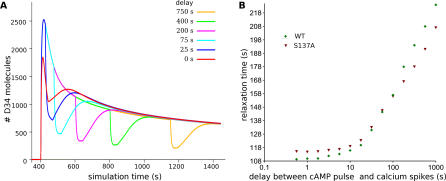
Effect of the Delay between cAMP and Calcium Stimuli (A) Time-course of D34* in model B, triggered by a pulse of cAMP, followed, after a variable delay, by a train of Ca^2+^ spikes. (B) Relaxation time of DARPP-32 response to calcium in function of the delay between cAMP pulse and Ca^2+^ spikes. Green diamonds represent the response of “wild-type” DARPP-32, while red triangles represent the response of a mutant without Ser137 phosphorylation.

After the Ca^2+^ activation ended, PP2B activity dropped, and total D34* quickly returned to the situation that followed the cAMP pulse. This translated into a transient increase of Thr34 phosphorylation, followed by a slow return to equilibrium. We used the relaxation time corresponding to the period between the minimum level and the transient maximum level of Thr34 phosphorylation reached during the relaxation phase as a characteristic of the “sharpness” of the response to glutamate signals (see Figure 5A). Figure 6A shows a superimposition of the different simulations, with a delay between cAMP and Ca^2+^ activation ranging from 0 to 750 s. Figure 6B shows the dependency of the relaxation time on the delay between the cAMP pulse and the calcium spikes. When the delay between the activation of both signals increased, the sharpness of Thr34 dephosphorylation decreased, showing that the coherence of the response between dopamine and glutamate signals depends on the time separating both activation pathways.

**Figure 7:**
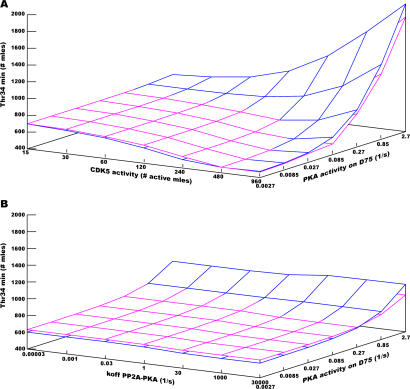
Cross-Sensitivity to the Inhibition of PKA by DARPP-32 and the Activity of CDK5 or the Stimulation of PP2A by PKA Values corresponding to model A are blue, while values corresponding to model B are magenta. (A) Cross-sensitivity to the inhibition of PKA by DARPP-32 and the activity of CDK5. Note the inverse relationship between CDK5 activity and Thr34min for strong inhibition of PKA (low kcat) while the relationship is reversed at weak inhibition. (B) Cross-sensitivity to the inhibition of PKA by DARPP-32 and the stimulation of PP2A by PKA.

**Figure 8:**
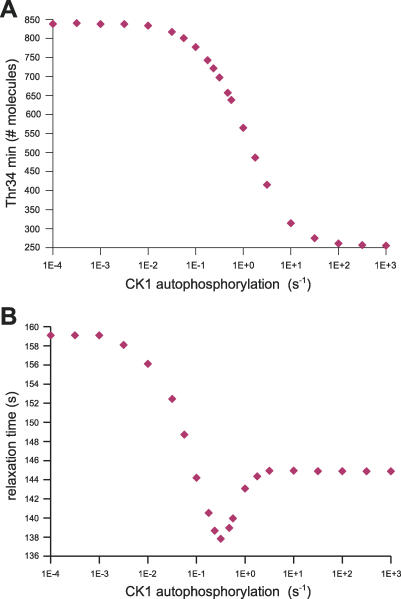
Sensitivity to the Auto-Phosphorylation Activity of CK1 (A) Sensitivity of Thr34min to the autophosphorylation activity of CK1. The maximal values of the *x*-axis correspond to a very fast auto-inhibition of CK1, with effects identical to a Ser137Ala mutation, see red curve on Figure 10A. (B) Sensitivity of the relaxation time after calcium signal (the “sharpness” of the response) to the autophosphorylation activity of CK1.

### Sensitivity Analysis

To analyse the robustness of the models to various parameters, we examined two characteristics of the variation of D34 in response to Ca^2+^ after cAMP: (i) the minimum level of Thr34 phosphorylation, characterising the “amplitude” of the response to Ca^2+^; and (ii) the relaxation of the response, as defined in the previous experiment. We chose these aspects of the response as a molecular indicator of the interaction between DA and glutamate to assess the importance of various factors in the regulation of DARPP-32. We ran sensitivity analysis experiments, studying the response of the models to the double perturbation, changing one or two parameters at a time. Below we present the result of this analysis for several parameters that displayed interesting behaviours. Those parameters are the activity of CDK5, CK1 auto-inhibition rate, the activation of PP2A by PKA, and the concentration of DARPP-32. We also evaluated the role of the inhibitory effect of D75* on PKA. In vitro, D75 inhibits phosphorylation of DARPP-32 on Thr34 by PKA. However, the level of this inhibition is not quantitatively known, either with D75 or D75:137 forms. Our sensitivity analyses were conducted at different levels of PKA inhibition, by varying the values of kcat8 and kcat21, the catalytic constants of phosphorylation by PKA of Thr34 on D75 and D75:137, respectively (see Table 1).

#### Role of CDK5.

DARPP-32-linked signalling pathway being thought to depend on the balance between Thr34 and Thr75 phosphorylations, we studied the role of CDK5 activity, which is responsible for Thr75 phosphorylation. Both models A and B showed similar behaviours. However, these behaviours depended on two parameters: the level of activity of CDK5 and the level of inhibition of PKA by D75* (Figure 7A). When PKA inhibition was high or complete (kcat8 and kcat21 ∼ 0), that is, the default case, Thr34min decreased when CDK5 activity increased. When CDK5 activity was high, Thr34min decreased when kcat8 and kcat21 decreased. The explanation comes from the functional equivalence, as far as PKA regulation is concerned, between an increase of CDK5 activity (more D75*) and a decrease of kcat8 and kcat21 (stronger inhibition of PKA by D75*). In contrast, when PKA inhibition was poor (kcat8 and kcat21 ∼ 2.7, that is, the same value as for the other forms of DARPP-32), then Thr34min increased with CDK5 activity. This apparent contradiction can be explained by the effect of Thr75 phosphorylation on the dephosphorylation of Thr34 by PP2B, which counteracted the effect of PKA. With little inhibition of PKA, an increase of D75* resulted in an increase of D34*. Hence, with an intermediate inhibition of PKA by D75*, the sensitivity of the models to CDK5 activity is a curve showing a minimum. Very low or high values of CDK5 activity similarly reduce the response to glutamate signals. In the former case, there is not enough D75* to inhibit PKA, and in the latter, the concentration of D75* is sufficient to trigger its phosphorylation on Thr34, despite the low activity of PKA.

#### Role of PP2A.

We also examined the sensitivity of the system to the phosphorylation of PP2A by PKA that increases its dephosphorylation activity on Thr75 (Figure 7B). Interestingly, no dramatic change was observed in either of the models, Thr34min and relaxation times hardly displaying any changes. When the stimulation of PP2A by PKA decreases (i.e., when koff33 and koff55 increase), the dephosphorylation of Thr75 diminishes, and therefore the inhibition of PKA by D75* increases. That should result in a lower Thr34min. However, the robustness of the coupling PKA–PP2A in our model can be explained by a segregation effect, the decrease of complexes PKA–D75* being compensated by more PKA–PP2A complexes. Therefore, PP2A effectively acts as a competitive inhibitor of PKA for DARPP-32.

#### Role of CK1.

To dissect out the role of CK1, we studied the sensitivity of the Ca^2+^ response to the catalytic rate of CK1 autophosphorylation (Table 1, kcat30). When the catalytic constant of this reaction increased, we observed an increase of the Ca^2+^ effect on Thr34 phosphorylation, that is, a decrease of Thr34min (Figure 8A). This was expected since the auto-inhibition of CK1 led to decreased Ser137 phosphorylation, and in turn less inhibition of Thr34 dephosphorylation by PP2B. However, we also observed an unexpected and unusual pattern for the sharpness of the response. Indeed, the relaxation time reached a minimal value at a particular catalytic rate (Figure 8B). This rate is very close to the one chosen as the default, based on a completely different rationale, that is, the minimal level of double D34:75 phosphorylations. Both models A and B displayed a similar sensitivity pattern, correctly reflecting the independence of Ser137 and Thr75 phosphorylations. Moreover, the auto-inhibition of CK1 is not affected by a change in PKA inhibition mediated by D75*. This is expected since CK1 inhibition and activation depends only on PP2B activity.

#### Influence of DARPP-32 concentration.

**Figure 9:**
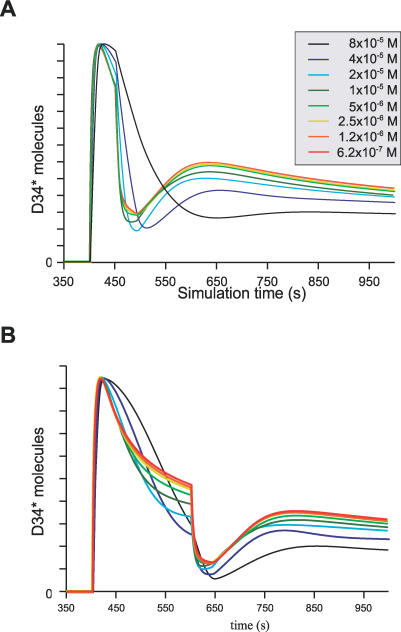
Dependency of the Signal Integration on the Concentration of DARPP-32 Same simulation paradigm as the one depicted in Figure 5, but with different concentrations of DARPP-32, all the other parameters being conserved. Only D34* of model B is plotted. While the *x*-axis remains the same for all time-courses, the *y*-axis is scaled to superpose all the traces. The vertical scaling is roughly linear, that is, a 2-fold increase between successive values of DARPP-32. (A) Calcium spikes started 50 s after the pulse of cAMP. (B) Calcium spikes started 200 s after the pulse of cAMP.

The concentration of DARPP-32 has been measured by crude biochemical approaches. In the striatum, it is thought to be in the range from micromolar to tens of micromolar. To evaluate the effect of DARPP-32 concentration on its function, we ran simulations with concentrations varying from 0.6 micromolar to 80 micromolar. Surprisingly, the time-course of D34* after a pulse of cAMP and calcium spikes stayed very similar across all concentrations (Figure 9). The absolute values were linearly scaled (twice more DARPP-32 gave twice more D34*), but the shape remained qualitatively the same. Between 0.6 micromolar and 10 micromolar, the time-courses were almost identical. At higher concentrations, the decay of D34* became slower, as was the recovery from calcium signals. More importantly, the ratio between the minimal D34* reached after calcium signals and the maximal D34* reached after a cAMP pulse stays constant (about 10%) whatever the concentration of DARPP-32.

**Figure 10:**
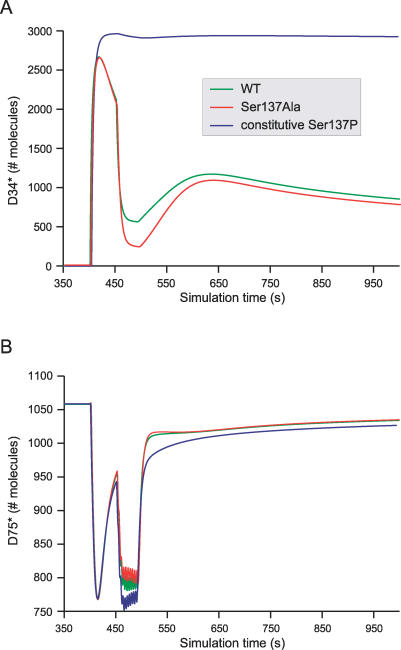
In Silico Site-Directed Mutagenesis of DARPP-32 Same simulation paradigm as the one depicted in Figure 5, but describing the predicted behaviour of mutants by model B. Wild-type DARPP-32 species are represented in green, Ser137Ala in red, and constitutive Ser137P in blue. (A) D34*; (B) D75*.

### In Silico Site-Directed Mutagenesis

To study the role of Ser137 phosphorylation, we built alternative models mimicking mutants. By setting all the catalytic constants of CK1 on DARPP-32 to 0, we emulated the behaviour of a mutant that can bind to CK1, but cannot be phosphorylated, equivalent, for instance, to a serine 137 to alanine mutant. Not surprisingly, the main effect of this change was to enhance the effect of calcium on Thr34 dephosphorylation by suppressing the incoherent feedforward loop through PP2B/CK1/Ser137 (Figure 10A). The effect on Thr75 was almost nil, which is coherent with the absence of cross-talk between Thr75 and Ser137 regulations (Figure 10B). More interesting than the effect on Thr34min was the effect on the relaxation time. For very short delays (inferior to 50 s), the mutant presented longer relaxation time, while for longer delays, its responses were sharper (Figure 6B). Although this effect was not very large (about 10% at 1000 s), it results in a less-efficient detection of the delay between signals, where the inhibition of PP1 differs less when the delay between cAMP and calcium varies.

By setting up all the catalytic constants of PP2C on DARPP-32 to 0, we can emulate, after equilibrium, a mutant with constitutive Ser137 phophorylation. This mutant was a quasi-irreversible switch. Because Ser137 phosphorylation strongly inhibits Thr34 dephosphorylation, the effect of a cAMP pulse was long-lasting. Calcium spikes had little effect on the amount of D34*. The basal activity of PP2B decreased D34* very slowly. After three days, two-thirds of the DARPP-32 was still phosphorylated on Thr34. The effect of this mutant on Thr75 phosphorylation is probably due to the fact that PKA is not inhibited by D34:75, and therefore more PKA is available to activate PP2A and dephosphorylate Thr75.

## Discussion

### Model Building

We built the model using classical chemical kinetics. We are conscious that due to the small size of a spine, some molecules represented in our models were present in a relatively low number of copies. Random fluctuations could therefore have a non-negligible impact [43]. Similarly, it is obvious that the well-stirred assumption (homogeneous distribution of molecules) is false. Ideally, postsynaptic signalling should be modelled using stochastic discrete simulations [44]. However, the lack of quantitative data (microscopic constants, precise localization, etc.) renders such an approach very inaccurate except in very specific cases. In addition, molecules varying widely and reaching very low numbers where stochastic simulations would be really necessary, such as Ca^2+^, have a significant impact on the model only when their concentration is high, that is, when the relative noise is low. Note that in the case of Ca^2+^, the increase and decrease of concentration are very quick compared with the other reactions of the model (phosphorylations and dephosphorylations). As a consequence, the use of population-based stochastic approaches, such as Gillespie derivatives, would not have changed the conclusions drawn from the simulations.

In E-Cell3, enzymatic reactions may be modelled using various kinetic laws. We used two of them: the Briggs–Haldane [45] derivation of Henri–Michaelis–enten [46,47] uni–uni kinetics (BH), or Mass Action Law (MAL). We assumed all enzymatic reactions were irreversible and that the enzyme-product species were negligible (i.e., transitory). However, depending on the kinetic law used, the treatment of enzyme-substrate complex was different. In BH, the complex enzyme substrate is assumed to be in a quasi–steady state. Therefore, net velocity of the BH kinetics does not require explicit calculation of enzyme substrate. On the other hand, using MAL to model enzymatic reactions requires the explicit description of all the intermediate species of the reaction, and the computation of their quantities.

We first constructed our DARPP-32 models using BH as the kinetic law for all enzymatic reactions. The first simulation results we obtained using these models showed unexpected results. Mass conservation was not respected within cyclic reactions pathways. In particular, the sum of all DARPP-32 moieties was not constant.

To investigate this problem further, we studied a stripped-down version of the model, consisting of one cyclic reaction pathway, modelling the double-phosphorylation states Thr34 and Thr75. In this mini-model, containing eight reactions, the PP2A/CDK5 couple of enzymes acts on Thr75 phosphorylation, and the PP2B/PKA couple acts on Thr34 phosphorylation. We discovered that, using BH kinetics, modifying the activity of only one couple of enzymes, e.g., PP2B/PKA changing the phosphorylation state at Thr34, also led to a change in the phosphorylation state at Thr75 despite no change in activity of PP2A/CDK5, which should be actually unchanged according to the law of mass conservation. This discrepancy is explained by the nonlinearity of BH. Because of different initial conditions on the parallel reactions, the resulting fluxes are not the same. In other words, there is not the same proportionality between the rate of production and the substrate concentration. A similar problem was reported in models of the MAP kinase cascade [48]. Subsequently, we chose to construct the same minimodel using MAL as the kinetic law for all enzymatic reactions. This required all enzyme-substrate complexes to be described. Moreover, reactions constants (association, dissociation, and catalytic constants) had to be estimated from experimental data, and usually calculated from Briggs–Haldane kinetic data (Km and Vmax). Using MAL, we found that simulations gave accurate results, the behaviour of the model being the expected one.

### Effect of Parameter Modifications

When building a reasonably large model like the one presented here, one necessarily has to estimate many parameters. Although these estimates are based on rational thinking (orthologous reactions in other organisms, function of similar enzymes, computation based on physical constraints, etc.), it is nevertheless important to verify that the conclusions of the study do not depend too much on the modeller's guesses. In addition, robustness and fragility can shed light on various aspects of a systems function [49,50].

Sensitivity analysis to the auto-inhibition rate of CK1 shows there are specific values that optimize the sharpness of the response to a Ca^2+^ activation following a cAMP signal, and dramatic influence on the amplitude of Thr34 phosphorylation. This shows that the auto-inhibition of CK1 is an important parameter in the system, since it influences the dynamic of integration of dopamine signalling by DARPP-32. Interestingly enough, the “optimal” value, i.e., the value giving the sharpest response, happened to be very close to kcat = 1 s^−1^, that is, the value we chose as default (Figure 8B). This value was chosen by a completely different criterion, as the one giving the minimal amount of D34:75:* phosphorylated forms. One could hypothesize that the regulation of DARPP-32 is finely tuned to discriminate between Thr34 and Thr75 phosphorylation, therefore dissociating PP1 and PKA inhibition. This demonstrates that the intricacy of control elements leads to a tradeoff between parameters, and nontrivial optimisation of the response and integration of cAMP and Ca^2+^ signals.

Sensitivity analysis shows that models A and B are both insensitive to changes in PKA-dependent PP2A stimulation (Figure 7B). This means that increasing the dephosphorylation rate at Thr75 by means of PKA-stimulated PP2A does not significantly affect the kinetic of Thr34 phosphorylation response to glutamate signalling. This contrasts with the results of a change of CDK5 activity (Figure 7A) and can be explained by the fact that changing CDK5 activity affects the basal phosphorylation at Thr75, and therefore the inhibition of PKA by DARPP-32. As a consequence, the basal level of phosphorylation at Thr34 is highly affected. This confirms the experimental studies on the effect of increased basal CDK5 activity, which showed a decrease of the effect of (cocaine-induced) dopamine signalling on Thr34 [20]. Conversely, increasing the stimulation of PP2A by PKA does not significantly change the basal level of phosphorylation at Thr75, but only the Thr75 response to cAMP pathway activation, which changes its amplitude of dephosphorylation/phosphorylation. Thus, the effect on PP2A activity through PKA is more transient than a change of the balance CDK5/PP2A. Our results show the fine-tuning between the different targets of PKA: PP2A, PDE, and DARPP-32, due to the high connectivity of the system.

Nishi and colleagues [15] showed that in the case of dopamine stimulation, i.e., cAMP increase, D34 and D75 were mutually antagonistic. However, the same authors showed afterward [16] that in the case of a glutamate stimulation, D34 and D75 were decreased together. One of the outcomes of the simulation derived from our model is that DARPP-32 is actually not a bistable switch. Although PKA/PP2A/D75 form a positive-feedback loop, the basal activity of CDK5 and PP2B in our model precludes the establishment of a bistable state, DARPP-32 being unable to be locked at 0% D75 or 100% D34 in wild-type situation (the situation is different in the case of a constitutive D137 mutant). One condition that facilitates the emergence of bistability is the presence of high-order reactions in the feedback loop [51]. One possible mechanism of PP2A regulation by PKA is known. The potentiation of phosphatase activity is due to the phosphorylation of the PP2A regulatory subunit by PKA (it has been demonstrated for the B′′ family so far) [52]. There are several phosphorylation sites on the regulatory subunit, but we are not aware of any indication that they trigger cooperativity. As far as we can tell, any of the phosphorylations trigger the release of the catalytic subunit.

### DARPP-32 as a Robust Signal Integrator

When the quantity of DARPP-32 was changed in the model, by up to two orders of magnitude, we observed a linear scaling of the D34* response (Figure 10). The global time-course of the response to a cAMP pulse followed by calcium spikes was conserved, but more importantly the ratio between the maximal D34* after cAMP and the minimal D34* after calcium is conserved. At higher concentrations, the decay of D34* became slower as was the recovery from calcium signals. This is particularly visible for the highest concentration, 80 micromolar, of DARPP-32 (although it is to be noted that such concentrations are huge, and very unlikely, despite what was reported earlier). This slower behaviour does not affect qualitatively the relative effects of cAMP and calcium. Therefore, the interplay of cAMP and calcium signals is conserved even at different concentrations of DARPP-32. Knock-out mice were produced for DARPP-32 [20], but, unfortunately, no phenotypes were reported on heterozygous animals, making it impossible to evaluate a dose-effect.

The delay between the cAMP signals and the calcium spikes significantly affects the relaxation time after calcium-induced Thr34 dephosphorylation (Figure 6). This shows that the coherence of the response between dopamine and glutamate signals depends on the time separating both activation pathways. DA, through the production of cAMP, modulates the duration of the response to glutamate. In turn, this points to the role of DARPP-32 as a signal integrator, since the relief of PP1 inhibition is related to the integral of the decrease of D34* (the area contained in the “well” caused by the calcium spikes). Note that this relation is not simple. Indeed, Thr34min, that is, the remaining DARPP-32 able to inhibit PP1, depends on delay. Therefore, the inhibition of PP1 would be different even if the area under the curve was the same.

The delays studied ranged from a fraction of a second to several minutes, so as to cover interstimulus intervals observed in electrophysiological and behavioural paradigms [9,41,42].

The effect of the delay between cAMP and calcium signals is attenuated in a mutant Ser137Ala. DARPP-32′s function of signal integrator is therefore impaired, and the effect of calcium on the relief of PP1 inhibition should be more independent of the previous cAMP signals.

In none of the sensitivity analyses performed did we observe a meaningful difference between models A and B. Although we successfully reproduce the observation of Nishi et al. [16] that Thr75 was decreased in response to calcium, this has little effect on PP1 inhibition. If there is a physiological consequence, it is probably mediated by the relief of PKA inhibition and its effect on targets other than DARPP-32 (such as AMPA receptors).

The model should now be extended upstream and downstream. Some forms of adenylate cyclase and PDE present in the striatum are sensitive to calcium [32,53,54]. An explicit modelling of these enzymes is therefore needed. To integrate cAMP and calcium signalling with other signalling systems, one needs also to model the effect of DARPP-32 on PP1 targets, such as ERK [55]. Finally, calmodulin and calcium/calmodulin kinase II need to be modelled explicitly in order to take into account the complexity of calcium effects.

Phosphorylated by four different kinases, dephosphorylated by three different protein phosphatases, and inhibiting a kinase and a phosphatase, DARPP-32 is one of the hubs of neuronal signalling. Since all those enzymes are themselves regulated by various signalling pathways, DARPP-32 acts as a computing unit [56] that could serve as a switchboard, modulating PKA and/or PP1 activity according to a whole ordered set of inputs. However, contrary to what was sometimes suggested before, our simulations show that DARPP-32 is not a sharp Thr34/Thr75 molecular switch. Upon activation of the cAMP pathway, we observed a dramatic change in the phosphorylation state of DARPP-32, which leads to a high level of D34*. But the participation of Thr75 dephosphorylation is much less important than what we thought. On the contrary, in this case, the pool of unphosphorylated DARPP-32 plays a major role in the conversion of DARPP-32 into a potent inhibitor of PP1. Moreover, as simulated with model B and in accordance with Nishi et al. [16], activation of the Ca^2+^ pathway leads to a simultaneous dephosphorylation at Thr34 and Thr75. Thus, the activity of DARPP-32 is finely regulated by different factors.

Because of the intricacy of its regulations, DARPP-32 is a robust signal integrator that not only filters the cortico–striatal inputs based on the internal state, but does so in a timely manner. An elevation of dopamine decreases the PP1-mediated inhibition of glutamatergic potentiation. It does that not only by counteracting the level of DARPP-32 dephosphorylation, but also by shutting down this dephosphorylation, an effect depending on the recency of the dopamine signal. The full complement of DARPP-32 phosphorylations is needed to get the full extent of this effect.

## Materials and Methods

### Modelling and simulation software.

Modelling and simulation were performed using the E-cell system version 3 [57] release 3.1.103 (http://www.e-cell.org/). The models are provided under E-Cell native format and in the Systems Biology Markup Language as Datasets S1–S4. E-cell system is an object-oriented software suite for modelling, simulation, and analysis of large-scale complex systems such as biological cells. The simulation environment uses a variable-process model, where a variable represents a molecular species and a process represents the kinetic law which results in a change in the value of a variable (the quantity of a molecular species). Each process is attached to a stepper, which decides the iteration step and performs the calculation. E-Cell supports various steppers, such as differential equation solvers. A generic ODEStepper developed by Kazunari Kaizu was used for the elementary reactions. The idea is to combine different types of single-step embedded Runge-Kuttas, rather than the using multistep methods that have been the norm. The specific combination used, Radau-5 + Dormand-Prince 4(5)7M, is the only generally available solver of this type (Kouichi Takahashi, personal communication), and is thought to be the best setting for computational cell biology problems by E-Cell developers. XPP-Aut [58] version 5.6 (http://www.math.pitt.edu/~bard/xpp/xpp.html), was also used to quickly test specific features of the models. The SBML versions of the models were tested on SBMLodeSolver (http://www.tbi.univie.ac.at/~raim/odeSolver/) directly or via CellDesigner (http://www.celldesigner.org/) and COPASI (http://www.copasi.org/). Simulations were performed on Intel-based computers under GNU/Linux, either on a monoprocessor desktop or a PC Farm at the European Bioinformatics Institute.

### Reaction parameters.

Reactions were modelled using either a mix of Briggs–Haldane and MAL processes, or solely with MAL processes. In the latter case, the enzymatic reactions were decomposed into three elementary steps. Association (k_on_), dissociation (k_off_), and catalytic (k_cat_) constants were usually calculated from published kinetic constants, retrieved from BRENDA (http://www.brenda.uni-koeln.de/), from DOQCS (http://doqcs.ncbs.res.in/), or taken from other published models [29,59]. Elementary constants were obtained from K_m_ using empirical methods.

Other parameters were estimated to match concentrations of the various DARPP-32 species observed in vivo [9]*.* All the parameters are listed in Table 1.

### Pathway activation.

cAMP and Ca^2+^ perturbations were performed using Python scripting, using the E-cell system API. cAMP input was realised by injecting a fixed amount of cAMP molecules into the system at one time. Ca^2+^ inputs were simulated by repeatedly increasing the calcium constant influx over 2 s, separated by 2 s at basal levels, triggering a series of short peaks. The basal constant influx is balanced by a constant decay of Ca^2+^ ions, to simulate re-uptake in the endoplasmic reticulum and buffering by calcium-binding proteins. All the initial conditions are listed in Table 2.

## Supporting Information

Dataset 1 Fernandez DARPP Model A SBML (XML 91 KB)

Dataset 2 Fernandez DARPP Model B SBML (XML 11 1KB)

Dataset 3 Fernandez DARPP Model A E-Cell Format (XML 80 KB)

Dataset 4 Fernandez DARPP Model B E-Cell Format (XML 100 KB)

## Abbreviations

CDK5: cyclin-dependent protein kinase 5, or, cell division protein kinase 5
CK1: casein kinase 1
CK2: casein kinase 2
DA: dopamine
DARPP-32: dopamine and cAMP-regulated phosphoprotein of 32kDa
MAL: Mass Action Law
PDE: phosphodiesterase
PKA: cAMP-dependent protein kinase
PP1: protein phosphatase-1
PP2A: protein phosphatase-2A
PP2B: protein phosphatase-2B
PP2C: protein phosphatase-2C
